# Introducing *Neurotrauma Reports*

**DOI:** 10.1089/neur.2020.28999.hb.editorial

**Published:** 2020-07-23

**Authors:** Helen M. Bramlett

**Affiliations:** Department of Neurological Surgery, University of Miami Miller School of Medicine, Miami, Florida, USA.

We are pleased to announce the launch of *Neurotrauma Reports*. This new, open access journal will serve as the companion journal to *Journal of Neurotrauma*. We will publish articles that may be more limited in scope or content that focus on a niche patient population. Additionally, as rejection rates have risen for the *Journal of Neurotrauma*, *Neurotrauma Reports* will be a valuable conduit for those articles that warrant publication as well. Authors will reach a wide audience as *Neurotrauma Reports* will be available online with no required subscription. As the competitive nature of science expands, publishing in a reputable open access journal is crucial for maintaining research integrity.

I am extremely excited to serve as editor-in-chief of *Neurotrauma Reports*. I will continue to maintain a close relationship with the *Journal of Neurotrauma* by remaining on the editorial board. Ms. Jean Ann Sanford will also extend her role as managing editor of *Journal of Neurotrauma* to include *Neurotrauma Reports.* Additionally, the current *Journal of Neurotrauma* editorial board has been offered the opportunity to serve on the editorial board of *Neurotrauma Reports* as well. These three points emphasize the continuity of operations for the new journal. My vision for this journal is to provide the neurotrauma field with not only another avenue for publishing original basic, translational, and clinical articles but also to reach individuals who have been impacted by central nervous system injury and disease with the availability of open access articles.

The mission of *Neurotrauma Reports* is to support basic, translational, and clinical neurotrauma publications to disseminate important information, increase education, and support awareness. Our goal is to publish quality articles for a global market.

Full submission instructions are available on the journal's website at www.liebertpub.com/neur To serve as a volunteer peer reviewer, please contact hbramlett@med.miami.edu

Finally, we thank the members of the editorial board for participating in the launch of this new important journal. *Neurotrauma Reports* will allow us to advance the field of neurotrauma with unparalleled access to this expanding area of research.

